# *Streptococcus intermedius* Brain Abscess with Lung Abscess and Aortic Valve Endocarditis: A Case Report and Literature Review

**DOI:** 10.3390/idr15040045

**Published:** 2023-08-11

**Authors:** Francesca Gavaruzzi, Pierangelo Chinello, Giuseppe Cucinotta, Gianluigi Oliva, Alessandro Capone, Guido Granata, Samir Al Moghazi, Emanuela Caraffa, Fabrizio Taglietti

**Affiliations:** National Institute for Infectious Diseases “L. Spallanzani”, 00149 Rome, Italy

**Keywords:** *Streptococcus intermedius*, brain abscess, lung abscess, endocarditis, case report

## Abstract

*Streptococcus intermedius* is frequently associated with brain and liver abscesses, while pleuropulmonary infections are considered rarer. Even less frequent is the association of lung and brain abscesses due to this agent with infective endocarditis. We describe the case of a 40-year-old man complaining of cough, fever, and headache who was diagnosed with a brain abscess due to *S. intermedius*, a concomitant lung abscess, and aortic native valve endocarditis. He was treated with surgical drainage of the brain abscess and a 4-week course of intravenous ceftriaxone, followed by oral amoxicillin/clavulanate, obtaining healing of the lesions without relapse of the infection.

## 1. Introduction

*Streptococcus intermedius* is a gram positive, catalase-negative coccus belonging to the *Streptococcus anginosus* group (SAG), which is often referred to as the *Streptococcus milleri* group, including *S. intermedius*, *S. anginosus*, and *S. constellatus*. SAG bacteria have been detected in the mouth, the gastrointestinal and upper respiratory tracts, and the vagina [1,2].

*Streptococcus intermedius* has been frequently associated with brain and liver abscesses, in rare cases related to endocarditis or congenital heart diseases [3,4,5,6,7,8]. Pleuropulmonary infections by this agent are uncommon [9], but are increasingly recognized. Absolutely anecdotal is the association of lung and brain abscesses due to *S. intermedius* with endocarditis [8]. We describe the case of a 40-year-old man with a brain abscess due to *S. intermedius*, a concomitant lung abscess, and aortic native valve endocarditis.

## 2. Case Presentation

The patient was a 40-year-old male of Egyptian origin who had resided in Italy for about 15 years and worked as a bartender. He had no allergies, previous hospitalizations, or surgical interventions; furthermore, he denied smoking habits, drug abuse, or alcohol consumption. For about 20 days, he experienced persistent cough, asthenia, intermittent fever, intense headache, and worsening back and lumbar pain that was partially responsive to therapy with paracetamol and non-steroidal anti-inflammatory drugs. Due to the worsening of these symptoms, he went to the Emergency Department (ED) of a district Hospital, where his vital signs were as follows: blood pressure 140/80 mmHg, heart rate 105 bpm, respiratory rate 17 breaths per minute, oxygen saturation 97% in room air, body temperature 38 °C, and Glasgow Coma Scale 15. There were no focal neurological deficits or meningeal signs. On thoracic objective examination, vesicular murmur was normally transmitted throughout the pulmonary field with no pathological moist noises; heart tones were paraphonic with poorly assessable pauses. On abdominal objective examination, the abdomen appeared flat, treatable, and non-tender on deep palpation, with negative Blumberg’s and Giordano’s signs. No major lymph nodes were palpable. The results of the oral cavity examination in the ED were not reported. Blood tests showed neutrophilic leukocytosis (white blood cells 12,900/mmc; neutrophils 77.7%) and a slight increase in C-reactive protein (1.2 mg/dL). The antigen test for SARS-CoV-2 was negative. No blood cultures were collected at that time. A non-contrast cranial computed tomography (CT) scan revealed a necrotic-colliquative expansive lesion measuring 36 mm in diameter in the left subcortical occipital area, with an associated perilesional oedema. The patient started treatment with ceftriaxone 2 g intravenously (IV) and methylprednisolone 4 mg as anti-oedema therapy.

**Figure 1:**
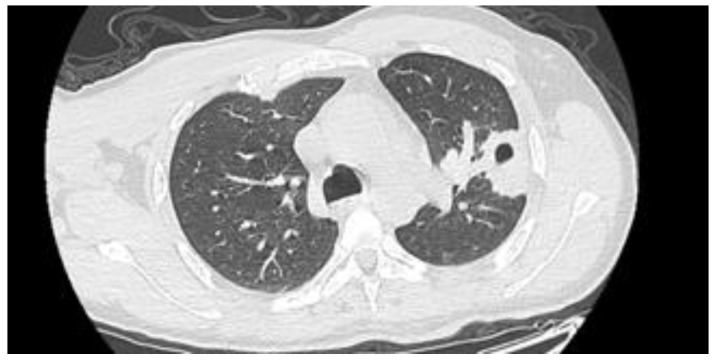
Chest CT scan showing a consolidated area with a thick-walled cavity lesion in the left lung lower lobe.

**Figure 2:**
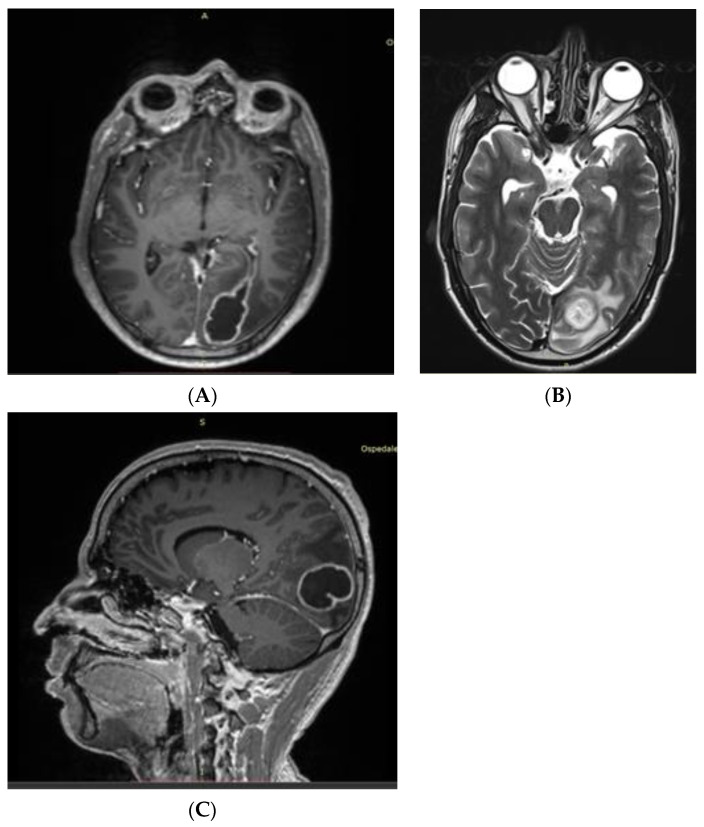
(**A**) First axial contrast-enhanced T1-weighted NMR showing an oval-shaped enhanced lesion in the left occipital lobe with ipsilateral ventricular communication. (**B**) T2-weighted NMR showing ventricular communication of the lesion with T2 hyperintense fluid collection. (**C**) T1-weighted sagittal view of the abscess.

He was then transferred to the ED of a second-level Center with Neurosurgical facilities, where a contrast-enhanced chest CT scan showed a pleural-based consolidative lesion in the left lung lower lobe measuring 63 mm in diameter, associated with a 16 mm internal cavity (Figure 1). The patient was admitted to the Oncologic Pneumology department. After an Infectious Diseases consultation, the antibiotic therapy was modified to include meropenem 2 g three times/day (TID) and vancomycin 500 mg four times/day. QuantiFERON-TB (QTF) Gold Plus and blood cultures resulted in negative results. A nuclear magnetic resonance (NMR) scan (Figure 2) confirmed the abscess nature of the brain lesion and revealed its communication with the trigone of the left lateral ventricle, along with perilesional oedema.

He was then transferred to the Neurosurgery department, where a bronchial aspiration was performed for direct microscopic examination, culture, and DNA testing for Mycobacterium tuberculosis complex, non-tuberculous mycobacteria, and Aspergillus. A full-spine NMR scan was also conducted but was negative for focal lesions. Pending microbiologic results, considering the possible tuberculous etiology of the disease, empirical combination therapy with linezolid 600 mg twice daily, meropenem 2 g TID, rifampicin 600 mg once daily (QD), isoniazid 300 mg QD, ethambutol 1200 mg QD, and levofloxacin 750 mg QD was started; dexamethasone 6 mg TID was also administered as suggested in the therapy of tuberculous meningitis [10].

Subsequently, a left occipital parasagittal craniotomy procedure was performed, resulting in drainage of the abscess and partial removal of the abscess capsule, along with sampling of biological material. The culture of the cerebral abscess samples resulted in a positive result for *Streptococcus intermedius*, which is sensitive to ampicillin, ceftriaxone, clindamycin, teicoplanin, and vancomycin. Thus, the patient started targeted therapy with ceftriaxone (2 g IV BID) and dexamethasone (12 mg IV BID). Following the QTF and PCR results, which tested negative for mycobacteria and fungi from bronchoalveolar lavage and from brain abscess specimens, the previous anti-tubercular therapy was discontinued. 

The patient was then transferred to our Infectious Diseases Center in stable and afebrile conditions. During the hospitalization, a transthoracic echocardiogram was performed to rule out heart valve involvement. An image with an uncertain interpretation was noticed in the aortic valve, so, in agreement with the cardiologists, this lesion was further investigated. A transesophageal color Doppler echocardiogram (TEE) was performed, revealing an aortic valve thickening with a prolapsing attitude of the right cusp and a nodular hyperechogenic image between the right and left cusps, along with mild valve insufficiency. The nodule was 8 × 6 mm in diameter without echocardiographic features suggesting a risk of embolization, possibly because embolization had already occurred. TEE was done after the diagnosis of a cerebral abscess and after 2 weeks of antibiotic therapy. There were no signs of left ventricular dilatation. An ultrasound scan of the abdomen was negative for embolic lesions. According to the 2023 Duke-ISCVID criteria [11], this case can be considered a definite endocarditis according to clinical criteria, as one major criterion (TEE positive) and three minor criteria (fever, cerebral abscess, positive culture from an embolus) were concomitant. Due to the presence of multiple carious lesions, a dental CT scan was performed, showing periapical bone resorption at tooth 14 in the upper arch and a large carious lesion at tooth 36, accompanied by an inflammatory periapical bone resorption area.

**Figure 3:**
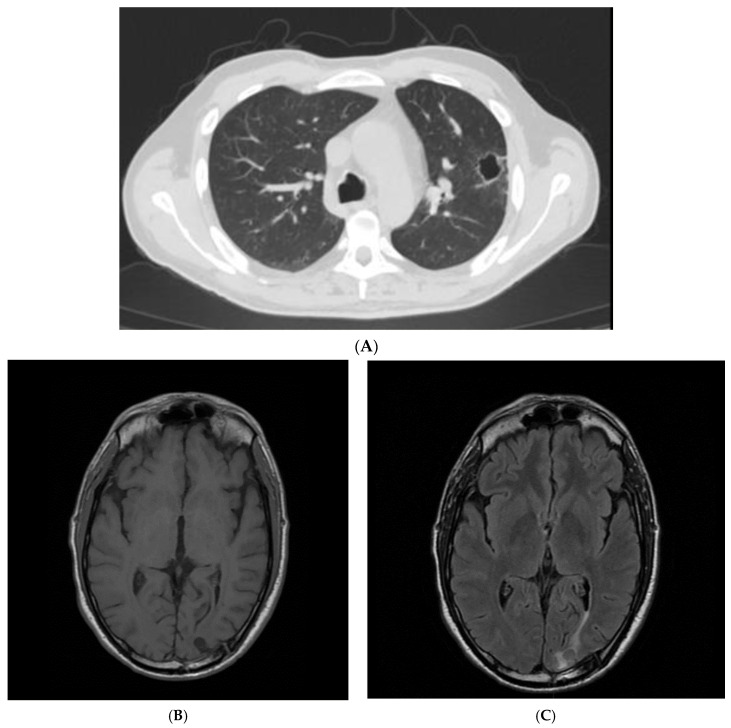
(**A**) A follow-up CT scan showing the reduction of the left lung lesion after a 4-week antibiotic course. (**B**) Follow-up T1-weighted brain NMR after a 4-week antibiotic course. (**C**) Follow-up T2-weighted brain NMR after a 4-week antibiotic course and surgical drainage.

The patient received a four-week course of ceftriaxone 2 g IV BID, resulting in progressive improvement of his clinical, laboratory, and radiological findings (Figure 3). He was discharged in good general condition with instructions to continue oral therapy with amoxicillin/clavulanate (875/125 mg TID) for another 10 days and a program of cardiological, neurosurgical, and dental outpatient visits. After a three-month follow-up, the patient is still in good health with no signs of infection relapse, as assessed in our outpatient clinic.

## 3. Discussion

*S. intermedius* is a known cause of brain abscesses and endocarditis. Research conducted by PubMed with the keywords “*Streptococcus intermedius*” AND “brain abscess” obtained 123 articles, while the keywords “*Streptococcus intermedius*” AND “endocarditis” yielded 57 papers. However, the association between *S. intermedius* endocarditis and brain abscess is rarely reported. In fact, using the keywords “*Streptococcus intermedius*” AND “endocarditis” AND “brain abscess”, we found 12 works, of which just 4 actually described cases of *S. intermedius* endocarditis with brain abscess [3,4,6,8], and 1 reported a case of *S. intermedius* brain abscess related to a patent foramen ovale [5] (Table 1).

Pleuropulmonary infections due to *Streptococcus intermedius* are also considered uncommon [12]; however, in recent years, their importance has been increasingly recognized. We reviewed the available medical literature by PubMed with the keywords “*Streptococcus intermedius*” AND “lung abscess”, “pleural effusion” and “pleural empyema” from 1993 to 2023, published in English. We found 34 articles meeting the inclusion criteria (Table 2), of which 4 described concomitant brain and lung abscesses, but none reported the presence of lung and brain abscesses due to *S. intermedius* associated with left sided endocarditis [1,2,8,9,12,13,14,15,16,17,18,19,20,21,22,23,24,25,26,27,28,29,30,31,32,33,34,35,36,37,38,39,40,41]. Only one paper [8] described the case of a lung and brain abscess associated with Chiari network endocarditis in the right heart.

*Streptococcus intermedius* is a Gram-positive, catalase-negative, nonmotile, and facultative anaerobe coccus that colonizes the mouth and the upper respiratory tract [2,33]. SAG streptococci are oral bacteria and may be unable to grow significantly on ordinary aerobic cultures; as a consequence, conventional cultivation may underestimate the role of these pathogens [32] in respiratory infections. The significance of anaerobes and oral bacteria in patients with community-acquired pneumonia and pleuritis has previously been reported [42,43]. In some cases, a 16S rRNA gene sequencing analysis of bronchoalveolar or pleural effusion specimens was able to identify pathogens that are generally difficult to isolate using ordinary cultivation methods [32].

Risk factors for *S. intermedius* pleuropulmonary disease include smoking, alcoholism, dental diseases, chronic obstructive pulmonary disease, malignant neoplasms, liver cirrhosis, and diabetes [9]. Our patient had dental lesions that could have caused bacteremia and aortic valve endocarditis, with a brain abscess as a hematogenous spread complication. The lung abscess could have been caused by aspiration or hematogenous spread. Teramoto et al. [44] reported that aspiration may contribute to the pathogenesis of pneumonia in elderly patients and that an increased age is associated with the risk of developing aspiration pneumonia. Given the young age of our patient, the aspiration in this case is less likely, whereas the hematogenous spread could have caused the lung abscess. Bacteraemic venous blood following the venous draining system to the right ventricle of the heart is pumped into the pulmonary arteries, the capillary network of alveoli, and parts of the visceral pleura. Indeed, several works describe the simultaneous occurrence of brain abscess, and lung abscess or pleural empyema [29]. In their recent work, Dyrhovden and coll. suggest that facultative and anaerobic oral bacteria, able to spread by deoxygenated venous blood to establish purulent infections in brain tissue, could also be able to reach and establish pyogenic infections in the lung parenchyma or pleural cavity [29].

*S. intermedius* is reported as a causative pathogen in only 2–5% of cases of bacterial pneumonia but in 13–44% of pulmonary abscesses/empyema [28]. *S. intermedius* infections are commonly associated with abscess development. Virulence factors contributing to tissue invasion and abscess formation by this agent have recently been described by Issa and coll [45] and include antigens I/II surface proteins; hydrolytic enzymes such as hyaluronidase, chondroitin sulfatase, and deoxyribonuclease; biofilm formation and defensive genes to oppose the human immune system, such as superantigens that cause lymphocyte apoptosis; the polysaccharidic capsule formation; the genes of the *Streptococcus* Invasion locus system; the intermedilysin, which initiates pore complex formation in host cell membranes, and the sialidase A that contributes to pathogenicity by controlling interbacterial communication and host-bacterial interactions. Abscess drainage and surgery should be considered on a case-by-case basis in order to successfully achieve adequate source control of the infection.

The major strengths of this case include the involvement of many different specialists in the management of this complex bacterial infection (ED clinicians, neurosurgeons, pneumologists, cardiologists, radiologists, infectious disease physicians, bacteriologists, and dentists) and the diagnostic work-up leading to the identification of the probable source of infection (dental lesions) and the relevant complications that followed. One important limitation is the lack of blood cultures drawn on hospital admission, which could have further strengthened the diagnosis.

## 4. Conclusions

In summary, we described a case of brain abscess caused by *S. intermedius*, associated with lung abscess and aortic valve endocarditis, in a young patient with dental disorders. In addition to being a well-known cause of brain abscesses, *S. intermedius* is increasingly recognized as a causative agent of pleuropulmonary disease.

## Figures and Tables

**Table 1:** Articles describing *S. intermedius* brain abscesses associated with endocarditis or PFO.

Article	n. of Patients	Sex	Age (y)	Infection Site	Outcome
Carrena et al., 2018 [8]	1	M	61	Lung, brain, Chiari network endocarditis	Recovered
Honnorat et al., 2016 [4]	1	M	56	Brain, endocarditis on surgical patch	Recovered
Syros et al., 2011 [5]	1	M	20	Brain; concomitant PFO	Recovered
Nakaya et al., 1998 [6]	1	M	60	Brain, mitral valve endocarditis	Recovered
Melo et al., 1978 [3]	1	M	69	Liver, brain and presumptive endocarditis	Death

PFO—patent foramen ovale.

**Table 2:** Articles describing lung abscesses and/or pleural infections due to *S. intermedius*.

Article	n. of Patients	Sex	Age (y)	Infection Site	Outcome
Shinzato et al., 1995 [1]	9	M = 8 F = 1	Mean 61.3	Lung, pleura	NA
Noguchi et al., 2015 [2]	14	M = 10 F = 4	Mean 77.3	Lung/pleura	Recovered = 12 Death = 2
Bueno et al., 2023 [9]	1	F	25	Lung	Recovered
Erne et al., 2010 [12]	1	M	61	Lung, brain	Recovered
Jerng et al., 1997 [13]	17	NA	NA	Lung/pleura	NA
Chandy et al., 2001 [14]	1	M	21	Blood, lung, frontal sinus, epidural abscess	Recovered
May et al., 2010 [15]	1	M	53	Lung	Death
Van Laren et al., 2011 [16]	1	F	29	Blood, lung, genital	Recovered
De Cruif et al., 2012 [17]	1	F	63	Lung, dental abscess	Recovered
Trabue et al., 2014 [18]	1	M	36	Lung, brain	NA
Maeda et al., 2012 [19]	1	F	46	Chest wall abscess, pleura	Recovered
Armendariz-Guezala et al., 2017 [20]	1	M	33	Brain, lung	Recovered
Sakurai et al., 2020 [21]	1	M	80	Pleura, iliopsoas abscess	Recovered
Carrena et al., 2018 [8]	1	M	61	Lung, brain, Chiari network endocarditis	Recovered
Fujihara et al., 2021 [22]	1	M	64	Lung, pleura	Death
Tasleem et al., 2021 [23]	1	M	54	Lung, pleura	Death
Manasrah et al., 2021 [24]	1	M	54	Lung, pleura, vertebrae, and discitis	Recovered
Nakagawa et al., 2022 [25]	1	M	6 mo	Lung, pleura	Recovered
Christensen et al., 1993 [26]	1	M	56	Lung	Recovered
Patail et al., 2020 [27]	1	M	30	Lung, pleura	NA
Takahashi et al., 2019 [28]	1	F	83	Pleura	Recovered
Dyrhovden et al., 2019 [29]	16	NA	NA	Pleura	NA
Cobo et al., 2018 [30]	9	M = 7 F = 2	Mean 63.9	Lung, pleura	Recovered = 8 Death = 1
Crespo Valades et al., 2005 [31]	1	M	60	Pleural effusion, subphrenic abscess	Recovered
Noguchi et al., 2014 [32]	1	M	79	Lung, pleura	Recovered
Hannoodi et al., 2016 [33]	1	F	52	Lung, pleura	Recovered
Mautner et al., 2000 [34]	1	M	80	Lung, pleura	Recovered
Huang et al., 2022 [35]	1	M	10	Lung, pleura	Recovered
Kurkowski et al., 2022 [36]	1	M	39	Liver, pleura, blood	Recovered
Jud et al., 2019 [37]	1	M	59	Lung, pleura	Recovered
Lescan et al., 2013 [38]	1	M	74	Lung, pleura, epidural abscess	Recovered
Stelzmueller et al., 2006 [39]	2	NA	NA	Pleural empyema	Recovered
Iskandar et al., 2006 [40]	1	NA	NA	Pleural empyema	NA
Lau et al., 2002 [41]	1	M	32	Pleural empyema, blood	Recovered

NA—not available; mo—months.

## Data Availability

The data presented in this study are available upon request from the corresponding authors.
